# A Paradigm Shift in Managing Carotid Artery Disease Associated with Coronary Artery Bypass Grafting

**DOI:** 10.21470/1678-9741-2025-0901

**Published:** 2025-05-23

**Authors:** Luciano Cabral Albuquerque

**Affiliations:** 1 Cardiovascular Surgery Department, Pontifícia Universidade Católica do Rio Grande do Sul - PUCRS, Porto Alegre, Rio Grande do Sul, Brazil

Carotid artery obstructive disease is highly prevalent and directly associated with advancing age. After the age of 60 years, an estimated 12% of men and 7% of women have moderate or severe asymptomatic carotid stenosis (CS) (≥ 50%)^[1]^. Likewise, the relationship between carotid and coronary artery disease has been extensively studied in recent decades. Consistent data indicate that the prevalence of ischemic heart disease ranges from 40% to 60% among patients with severe CS^[2]^. On the other hand, in patients undergoing evaluation for coronary artery bypass grafting (CABG), the rate of moderate or severe CS reaches 20%^[3]^.

For decades, CS was considered the primary cause of ischemic neurological events in the postoperative period of CABG, leading to the widespread practice of “prophylactic” carotid endarterectomy performed concurrently with CABG. However, contemporary evidence from multiple controlled studies has changed this paradigm, prompting a shift in management strategies.

In the treatment of carotid artery disease, intervention is primarily guided by the presence of focal neurological symptoms ipsilateral to the lesion and the degree of stenosis. For patients who have experienced a transient ischemic attack (TIA) or ischemic stroke within the past six months, the evidence supporting intervention is well established, dating back over three decades. Landmark trials, including the North American Symptomatic Carotid Endarterectomy Trial (or NASCET) and the European Carotid Surgery Trial (or ECST), demonstrated that patients with stenosis ≥ 50% benefit from carotid surgery, as long as they do not have comorbidities that limit five-year survival and the institution's perioperative risk of death and/or stroke is < 6% (level of evidence: 1a). Furthermore, carotid intervention is recommended within 15 days of diagnosis due to the high risk of recurrent cerebrovascular events^[4]^.

However, in neurologically asymptomatic patients, advances in optimized clinical treatment of atherosclerosis have significantly reduced the need for carotid intervention. The Asymptomatic Carotid Atherosclerosis Study (or ACAS) and the Asymptomatic Carotid Surgery Trial (or ACST) in the 1990s demonstrated a benefit of endarterectomy in patients with stenosis ≥ 60%, provided they had a survival expectancy of more than five years and a perioperative stroke or death risk of up to 3%. At the time, the available clinical treatment was associated with an annual stroke rate of 4% to 6%.

In recent years, however, the introduction of high-potency statins and aggressive low-density lipoprotein cholesterol management has lowered the annual stroke rate in asymptomatic carotid disease to approximately 2%, a risk comparable to or even lower than that of surgical intervention. Therefore, the current challenge lies in identifying which asymptomatic patients are at high risk for stroke. While neurological symptoms serve as a clear manifestation of plaque vulnerability, in asymptomatic patients some imaging characteristics can identify high-risk plaques.

Several studies have consistently shown that the presence of intraplaque hemorrhage on magnetic resonance imaging, ulceration, echolucency or increased plaque volume on Doppler ultrasound, silent cerebral infarction, and evidence of embolization on transcranial Doppler increase stroke risk by a factor of four to eight. Recognizing their prognostic value, these markers were incorporated into the intervention algorithm in 2018. Thus, the current indication for intervention in asymptomatic patients with carotid disease is stenosis ≥ 60% accompanied by one or more signs of vulnerability on the imaging methods aforementioned^[5]^.

Postoperative stroke remains a devastating complication after CABG, with high morbidity and mortality, occurring in approximately 3% to 5% of patients despite advancements in monitoring, anesthetic techniques, and cardiopulmonary bypass (CPB). For many years, carotid disease was considered the primary cause of post-CABG neurological events. However, evidence from several contemporary studies demonstrates that, while CS is associated with an increased risk of post-CABG stroke, > 80% of postoperative strokes occur in patients without any CS. Additionally, only 7% of all strokes occur in patients with plaques causing 50 - 99% obstruction^[6]^, a rate that rises to 20% in those with recent neurological symptoms^[7]^. These findings reinforce the current concept that the pathophysiology of post-CABG stroke is complex and multifactorial.

Beyond carotid obstruction, atheroembolic events may arise from aortic clamping, insertion and removal of the perfusion cannula, and construction of proximal bypass graft anastomoses. Prolonged CPB may lead to cerebral hypoperfusion, while the CPB circuit itself can trigger a systemic inflammatory response, resulting in neurological complications or cognitive impairment, especially in elderly patients. Furthermore, new-onset atrial fibrillation, which occurs in approximately 25% of patients postoperatively, increases the risk of cardioembolic stroke.

Among these factors, aortic manipulation is responsible for approximately two-thirds of post-CABG strokes and has been the target of prophylactic technical measures. Some strategies are now routinely incorporated into CABG protocols, such as preoperative imaging assessment of cases with high atherosclerotic burden, epiaortic echo-Doppler-guided cannulation, and/or partial non-clamping, or even the use of exclusively arterial grafts, without any aortic manipulation^[8]^.

Given these factors, the management of patients with carotid artery disease in the context of CABG has evolved significantly in recent years. Current evidence suggests that the primary indication for carotid intervention in CABG candidates is the presence of neurological symptoms related to carotid plaque in the last six months. In contrast, asymptomatic patients are generally not considered for carotid surgery, except in cases of severe bilateral disease. This evidence is largely supported by findings from the recent Coronary Artery Bypass Graft Surgery in Patients with Asymptomatic Carotid Stenosis Study (or CABACS) trial, which randomized 127 CABG candidates with asymptomatic CS ≥ 70% to undergo either CABG alone or combined surgery strategies. In a five-year follow-up, there was no significant reduction in death or stroke in the group undergoing CABG + carotid endarterectomy^[9]^. This finding, incorporated into the most recent guideline of the European Society for Vascular Surgery, supports the most recent recommendation against routine intervention in asymptomatic disease^[4]^.

**Fig. 1:**
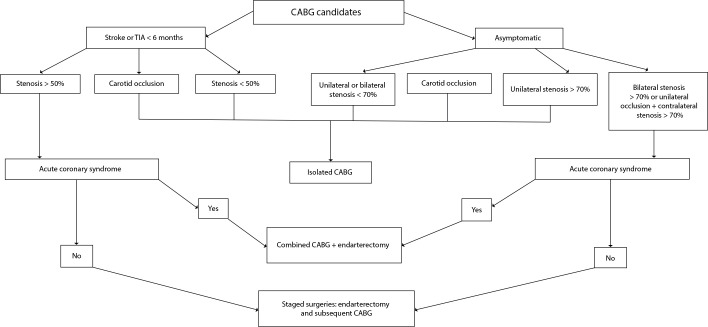
Carotid disease management strategies in the context of coronary artery bypass grafting (CABG). Adapted from Naylor et al.^[4]^. TIA=transient ischemic attack.

In patients with recent TIA or stroke caused by CS ≥ 50%, endarterectomy should be performed, either staged or concomitantly with CABG. Although studies comparing different strategies have shown conflicting results, it is well established that combined surgery increases the risk of death and permanent neurological deficit^[10]^. Therefore, the preferred approach in patients with stable angina is to perform carotid surgery first, followed by CABG. The only scenario that justifies simultaneous procedures is acute coronary syndrome, where both conditions are in a vulnerable phase, necessitating urgent intervention (Figure 1).

Finally, carotid stent angioplasty should be disregarded as an intervention option for carotid disease, unless there is an anatomical impediment to conventional surgery. This is due to the higher risk of periprocedural stroke compared to endarterectomy in this patient subgroup, as well as the need for dual antiplatelet therapy, which is undesirable in the context of myocardial revascularization.
